# Gaps in the Global Regulatory Frameworks for the Use of Artificial Intelligence (AI) in the Healthcare Services Sector and Key Recommendations

**DOI:** 10.3390/healthcare12171730

**Published:** 2024-08-30

**Authors:** Kavitha Palaniappan, Elaine Yan Ting Lin, Silke Vogel, John C. W. Lim

**Affiliations:** Centre of Regulatory Excellence, Duke-NUS Medical School, Singapore 169857, Singapore; elainelin@duke-nus.edu.sg (E.Y.T.L.);

**Keywords:** gaps in regulatory frameworks, Artificial Intelligence, healthcare services

## Abstract

Artificial Intelligence (AI) has shown remarkable potential to revolutionise healthcare by enhancing diagnostics, improving treatment outcomes, and streamlining administrative processes. In the global regulatory landscape, several countries are working on regulating AI in healthcare. There are five key regulatory issues that need to be addressed: (i) *data security and protection*—measures to cover the “digital health footprints” left unknowingly by patients when they access AI in health services; (ii) *data quality*—availability of safe and secure data and more open database sources for AI, algorithms, and datasets to ensure equity and prevent demographic bias; (iii) *validation of algorithms*—mapping of the explainability and causability of the AI system; (iv) *accountability*—whether this lies with the healthcare professional, healthcare organisation, or the personified AI algorithm; (v) *ethics and equitable access*—whether fundamental rights of people are met in an ethical manner. Policymakers may need to consider the entire life cycle of AI in healthcare services and the databases that were used for the training of the AI system, along with requirements for their risk assessments to be publicly accessible for effective regulatory oversight. AI services that enhance their functionality over time need to undergo repeated algorithmic impact assessment and must also demonstrate real-time performance. Harmonising regulatory frameworks at the international level would help to resolve cross-border issues of AI in healthcare services.

## 1. Introduction

**Figure 1:**
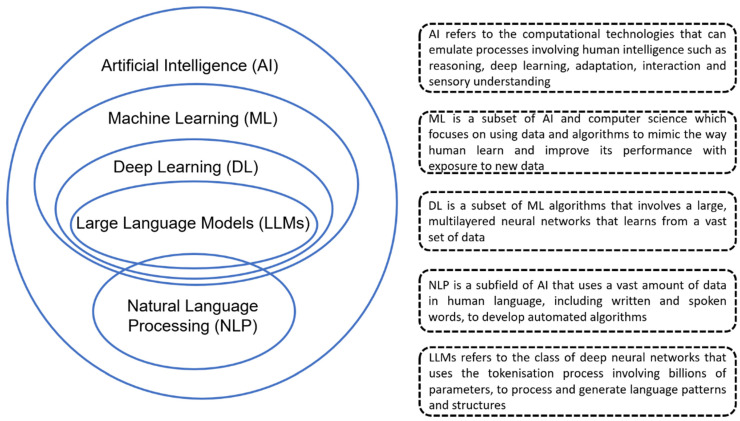
Classification of Artificial Intelligence (AI).

Artificial Intelligence (AI) refers to the capabilities of a machine to learn from experiences in the form of inputs by humans and perform human-like tasks [1]. A subset within AI is data-based algorithms, commonly referred to as machine learning (ML), where the machine can learn without being explicitly programmed and perform according to what it has learnt. Deep Learning (DL) is a subset of ML that involves the training of complex algorithms known as artificial neural networks (ANN) or deep neural networks (DNN) to perform brain-like reasoning and logical tasks. Within DL are Large Language Models (LLMs) that analyse large text datasets by computational methods referred to as natural language processing (NLP) and can answer questions in a conversational model (Figure 1).

**Figure 2:**
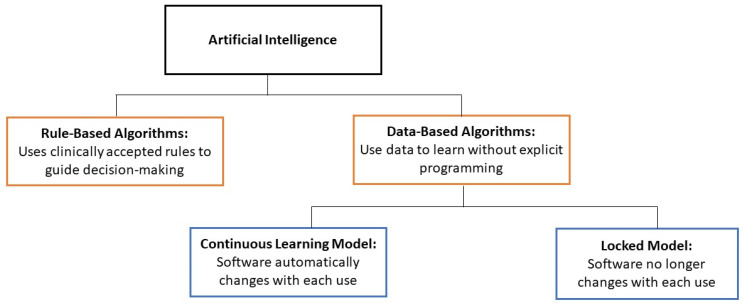
Categories of AI algorithms.

Based on functionality, AI can be divided into two categories: rule-based and data-based algorithms (Figure 2). Data-based algorithms are more commonly known as machine learning.

With its introduction into the healthcare sector in the 1970s, when the MYCIN system was developed at Stanford University to diagnose infectious diseases [2], AI has offered a range of straightforward and more radical opportunities that include the automation of administrative functions, supporting diagnosis through evidence-based clinical decision making and suggesting suitable treatments by analysing huge amounts of data within a short duration [3]. While the application of AI in healthcare is still relatively nascent, it has the potential to significantly improve patient health outcomes [4] and the well-being of healthcare professionals [3].

As with every new technology, AI presents its own risks and challenges which, if not adequately addressed, could impede the further adoption of AI technologies in healthcare. Governments around the world are faced with challenges in data security and protection, data quality, validation of AI algorithms, accountability and liability, and ethics [5]. Policymakers recognise that swift actions must be taken to mitigate the risks of AI technologies in the dynamic healthcare landscape through new regulatory guidelines. As AI technologies evolve, regulatory agility will be necessary to mitigate the risks and overcome the above mentioned challenges [6]. Regulating AI in the healthcare services sector presents unique challenges and complexities compared to other sectors due to the critical nature of healthcare, its ethical implications, and the direct impact on human lives.

This article aims to identify gaps in existing global frameworks for regulating AI in healthcare services and recommend approaches that could be adopted to address these gaps.

## 2. Methodology

A desk review was conducted to understand the existing regulatory landscape for the effective use of AI in healthcare services and the gaps in associated AI regulatory frameworks. The desk review focused on 4 broad questions:

- How is AI that is used in the healthcare service sector regulated across the world?
- What are the gaps in such regulations?
- How have these gaps been addressed by different countries?
- What are the unaddressed gaps?

Internet searches using the key words “healthcare AI regulations, legal framework, standards, guidelines, regulatory gaps, regulatory challenges and compliance strategies” were conducted on seven electronic databases, namely, EBSCO, Embase, PubMed, SCOPUS, ScienceDirect, Springer, and Web of Science. Google search and snowballing (screening all articles that cited the referenced paper) were also used to identify grey literature. Only articles pertaining to the use of AI in healthcare were included in this study and any other article that discussed about AI in other sectors were excluded. The articles from the database and internet search were studied and the relevant information extracted for this review. The gaps in the existing regulatory frameworks were grouped into five major themes based on the critical areas where regulatory gaps exist for healthcare AI.

## 3. Results and Discussions

The regulatory frameworks (both hard and soft laws) relevant to healthcare AI from seven jurisdictions, namely, the United States of America (USA), the United Kingdom (UK), Europe, Australia, China, Brazil, and Singapore were studied [7] and analysed for regulatory gaps. Some countries regulate AI under the ambit of Software as Medical Devices (SaMDs) while others regulate this separately using a risk-based approach, with provisions that include good machine learning practices, holistic life cycle approaches, and impact assessments for AI. The International Medical Device Regulators Forum (IMDRF) defines SaMD as “software intended to be used for one or more medical purposes that perform these purposes without being part of a hardware medical device”. Even though several countries incorporate AI-based Medical Devices (MDs) under SaMDs, there are considerable differences between the two. Unlike SaMDs that use locked AI models, AI-based MDs use algorithms that have the capability to work autonomously, learn continuously, and change their results over time based on new datasets encountered during the process. Thus, the risks of using such algorithms are higher than for conventional medical device software [8]. Due to the complex working system, there is a high possibility for the performance of an AI-based MD to differ to a considerable extent in the actual practice settings compared to the testing and learning environment that was used for the approval process [9].

Although the existing regulations are being applied to try and account for the increasing use of AI in healthcare, there are remaining regulatory gaps that need to be addressed to ensure the safety, efficacy, and ethical use of AI in the healthcare service sector. Specifically, the AI algorithms in AI-based MDs that can self-learn and adapt have the potential to introduce new and unknown risks that may supersede the risks initially identified by the developers and regulators. These therefore should be regulated separately to ensure patient safety and improve care [10].

**Figure 3:**
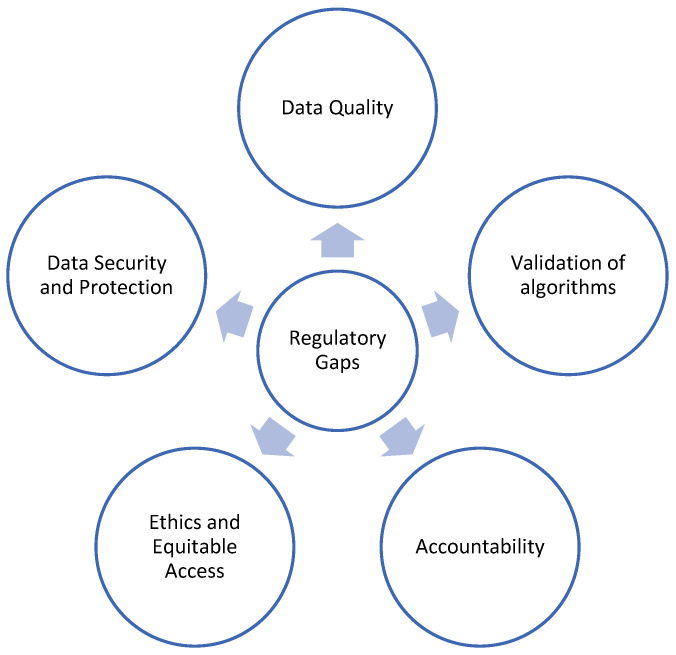
Regulatory gaps in AI for healthcare services.

The gaps in existing regulatory frameworks and potential recommendations to address these are discussed below in relation to the five major thematic issues of concern for the use of AI in healthcare services: (i) data security and protection, (ii) data quality, (iii) validation of algorithms, (iv) accountability, and (v) ethics and equitable access (Figure 3). While other issues such as interoperability, education, transparency, and legal liability are also important, they may either be subsumed within the identified themes or represent emerging areas that are gradually being recognised. The rationale for focusing on these five themes is their direct impact on the safety, effectiveness, fairness, and trustworthiness of AI in healthcare. 

### 3.1. Data Security and Protection

Data are fundamental for the development of AI applications and models as huge amounts of data are required to train such models. Most countries have data protection regulations but such policies are not uniform, resulting in vulnerabilities from the perspective of data security and protection. Data security and privacy are compromised by regulatory gaps and underlying issues in data anonymisation, data exportation, and informed consent, which are summarised in Table 1 and discussed in subsequent sub-sections.

#### 3.1.1. Anonymisation of Data

The exclusion of anonymous data from legislation such as the Health Insurance Portability and Accountability Act (HIPAA) of 1996 in the US, the General Data Protection Regulation (GDPR) in the European Union, the Personal Data Protection Act (PDPA) in Singapore, and the Privacy Act of 1988 in Australia raises issues about the security of data as the issues related to anonymous data are not addressed in these regulations. For example, HIPPA permits the disclosure of genetic information without consent for anonymous data and does not apply to all organisations. Private organisations [11] and most health apps are not covered under HIPPA [12,13]. However, with the advancement of technology, many studies have shown that it is quite possible to re-identify individuals from anonymised data and hence the privacy of the individuals is at stake in such cases [14].

#### 3.1.2. Data Exportation

Due to the lack of uniform data protection and regulatory frameworks across the world, many organisations that are involved in generating AI models try to utilise data gathered from countries with weak or no data protection regulations for various purposes such as research and development of new AI models. Hence, data exportation is prone to data insecurity and sufficient measures to protect such data obtained from countries with weak or no data protection regulations need to be factored in by the receiving entities, whether these are organisational leaders or regulators.

#### 3.1.3. Informed Consent

The patients and users of AI/ML applications need to be informed and must give consent when using these applications [10]. Patients should also have the option for the disclosure of their data, the intention for such disclosure, and how the data will be handled. However, this is challenging because users often do not completely understand the implications of their data usage in AI/ML applications.

#### 3.1.4. Recommendations

It is important for organisations handling patient data to ensure that their anonymisation processes are robust enough to prevent the possibility of re-identification, considering the means reasonably likely to be used either by the organisation itself or by any other person to identify the individual, directly or indirectly. Some techniques of data anonymization such as differential privacy that adds random noise to datasets to prevent the identification of individuals [15], homomorphic encryption that allows computations to be performed on encrypted data without decrypting it [16], data masking that involves modifying data to hide sensitive information while maintaining the utility of the data [17], K-anonymity, which ensures that each record is indistinguishable from at least k-1 other records regarding certain identifying attributes, and L-diversity and T-closeness, which ensure diversity and similarity, respectively, in sensitive attributes [18] could be explored. Stringent requirements such as robust de-identification standards, the requirement of regular risk assessments, strict access control policies, and the development of new privacy-preserving technologies should be set to minimise the risk of re-identification of patient data. For issues pertaining to data exportation, regulatory frameworks could factor in the principles such as defined and limited purposes and the possibility for patients to erase stored data or stop its further usage, irrespective of the source country of the data. When there is a need to reuse data for secondary purposes, it is necessary to balance individual autonomy, safeguards, and the public interest, especially when it involves healthcare data [19,20]. Blockchain technology can be used to create secure, decentralised data storage systems. By using smart contracts and cryptographic techniques, blockchain can ensure that healthcare data are accessed and shared in a secure and controlled manner [21]. Yet another method that can be explored would be the federated learning method that allows the model to learn from data without the need to centralise it, thereby preserving privacy [22].

### 3.2. Data Quality

**Figure 4:**
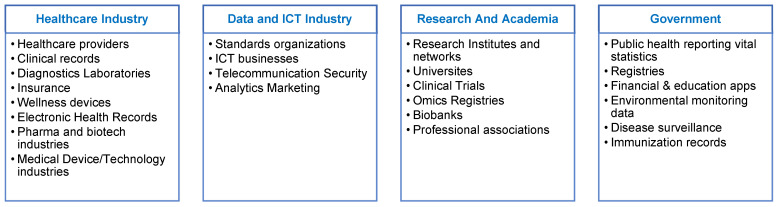
Sources of health data used by AI technologies.

Health data can be gathered from various sources. These could be from within or across different sectors, encompassing healthcare and infocomm technology, research and academia, and government (Figure 4).

#### 3.2.1. Bias in Data

The data with which the AI is trained needs to be unbiased. For healthcare, the training data needs to also factor in key demographic factors such as gender, age, race, and lifestyle markers for the data to be unbiased. This is not an easy task as bias may occur at one or multiple stages such as at the time of problem selection, data collection, outcome definition, algorithm development, and post-deployment. The danger of biased data is that it could lead to improper or wrong diagnosis or treatment, affecting a patient’s safety [23].

Data colonialism is a process by which governments, non-governmental organisations, and corporations claim ownership of and privatise data that are produced by their users and citizens. Such data colonialism has the risk of introducing bias in AI algorithms as they come from one specific group of people or country. Data colonialism is a common phenomenon that has been identified to affect the quality of data in many companies that train AI for health-related purposes [24].

The quality of data will also be affected by the context in which it is collected. A low income setting, for example, may have the following barriers to data collection: (i) language barriers; (ii) excessive burden on healthcare workers spending time on collecting data; (iii) lack of data for marginalised and vulnerable groups of people; (iv) people lacking trust in the government and thus withholding data; and (v) lack of education resulting in people providing wrong information or irrelevant data [6].

An AI model’s performance or output depends on the data input. If the input data are of high quality, the output will be more accurate, reliable, and valid. Data quality is impacted by how representative the data is of the population for which the AI is being developed. Thus, training an AI on the population for which the AI is going to be ultimately used is important.

#### 3.2.2. Representative Data

The collection and availability of useful and representative data remains a challenge for the training and validation of AI technologies such as ML models. Moreover, ML models could be applied to various data types such as images, speech, videos, and text. In a healthcare setting, there are large amount of textual data such as doctor’s notes and medication orders but also high imagery data such as CT scans and X-rays. The ability for the AI technology to extract critical information from such a large dataset is paramount in the improvement of diagnoses and decision making for patient treatments, which impact health service delivery and patient treatment outcomes. Hence, there is a need for health organisation leaders to recognise such issues and support the retraining and validation of ML models through the provision of real-world data that is diverse, balanced, and representative [25].

#### 3.2.3. Recommendations

It is important to ensure the quality of data by seeing how well four properties are fulfilled—(a) *variety* (structured and unstructured data from health data sources), (b) *volume* (obtaining sufficiently large amounts of health data), (c) *veracity* (level of trust in the data that stems from the accuracy and quality) and (d) *velocity* (the speed at which data are generated, collected, accessed, and processed) [26].

One way to address the issue of bias in training data is to disclose the attributes of the data, as is expected under the European Union’s AI Act. In this Act, all details about the training datasets used are required to be transparent. This includes information about the provenance of datasets, their scope, main characteristics, procurement and selection of data processes, labelling procedures for supervised learning, and data cleaning methodologies [27].

Policies need to include clear data quality standards and procedures such as data entry protocols, data validation rules, data quality metrics, data correction processes, standardised forms and templates, real-time validation checks, automated data cleaning tools, and effective feedback mechanisms.

### 3.3. Validation of Algorithms

The regulatory bottlenecks, trustworthiness, and transparency issues surrounding the application of AI in healthcare services are essentially concerns about the scientific validity of the analytical and clinical performance of AI systems. While the EU has explicitly stated requirements under the GDPR that machine learning algorithms are required to be able to explain their decisions [28], given the complexity and scale of LLMs, it is challenging for developers and policymakers to fulfil this. The key challenges and underlying issues for validating the AI algorithms are summarised in Table 2.

#### 3.3.1. Interpretability and Explainability

The difficulty in validating AI systems is due to the complexity of the AI algorithms and a lack of understanding how an algorithm has reached the conclusion in determining a particular diagnosis or predicting the suitable treatment for the patient. This “black box” phenomenon is an inherent aspect of the nature of AI-based prediction models [32].

In healthcare services, a patient’s health record data and indicators span across time and are interconnected in multiple ways to form a non-linear relationship [32]. Hence, the predicted results by AI models are not easily interpretable by the end users, including physicians and other healthcare professionals [33]. Enhancing the interpretability and explainability of AI models is imperative to promote the acceptance and trust of AI in healthcare services by healthcare professionals and helping to resolve regulatory bottlenecks by regulators and policymakers [34].

#### 3.3.2. Recommendations

Explainable Artificial Intelligence (XAI), which is a set of features that explain how the AI model constructed its prediction, has been widely used for the validation of AI algorithms [3].

An example of an XAI technique is the layer-wise relevance propagation that generates saliency maps, highlighting relevant inputs responsible for the results or recommendations generated within the neural networks [35]. Saliency maps refer to the visual results produced from the AI algorithms and are one of the simpler forms of explainable AI techniques. This visualisation of predicted results from AI models is crucial for the explainability and interpretability of AI in the healthcare services setting to aid physicians in their decision making for the diagnosis of patients [36].

On the other hand, the effectiveness, efficiency and satisfaction related to a causal understanding of a decision, commonly known as causability, is essential along with the explainability of the AI system. Explainability and causability need to be mapped to bridge the gaps between machine and human decisions made by the healthcare professional [37].

Interdisciplinary collaboration between AI developers and healthcare professionals is crucial for improving AI algorithm validation. By combining technical expertise with clinical insights, this partnership ensures that algorithms are not only technically sound but also clinically relevant and safe. Healthcare professionals provide valuable context, identify practical challenges, and validate outcomes, while AI developers bring advanced analytical tools, enhancing the robustness and applicability of AI solutions in real-world healthcare settings.

Furthermore, policymakers need to be aware that AI technologies are not one-sized solutions that could be used on digital healthcare systems across the healthcare continuum. With the adaptive learning capability of AI, it is necessary for regulators to address the adaptive algorithms that can adjust parameters or behaviour based on the input data or performance on a specific task. One possible recommendation is to conduct preliminary pilot trials for the validation of AI applications for health services [25].

### 3.4. Accountability

Accountability refers to the obligation and capability to answer questions regarding decisions and/or actions [38]. The issue of where the accountability lies when an AI system commits an error is an ongoing topic of discussion among developers and policymakers globally.

#### 3.4.1. Legal Liability

Considerations on legal liability and adverse event monitoring are part of ensuring accountability for the use of AI in healthcare services. If the gap in accountability is not managed in a systematic manner, the lack of trust in AI in healthcare services will not be addressed, and medical practitioners and patients would fail to reap the benefits of AI technologies [39].

#### 3.4.2. Cross-Border Challenges

In terms of healthcare services such as telemedicine, the regulatory issue of accountability and liability rises when the two parties—the physician and the patient—are in different countries that are governed by different sets of regulations. This raises the question as to which law would be applicable when a medical malpractice claim arises [40].

#### 3.4.3. Recommendations

A recent study provided some recommendations to address the accountability and liability issues. The developer could be made liable for errors in the algorithm computation, the AI-trained health professional could be made liable if a mistake occurred while using the technology, and the hospital could be made liable if a particular AI technology was imposed on its healthcare professionals, irrespective of their views [41]. Yet another model could be the “no-fault compensation” model, which involves creating a fund to compensate patients for injuries caused by AI errors without attributing blame to specific parties. It aims to simplify compensation processes and encourage innovation. The Vaccine Injury Compensation Program (VICP) in the US serves as an analogy for this, where a fund compensates individuals adversely affected by vaccines without litigation [42].

With regard to cross-border challenges, establishing mutual recognition agreements for licencing and certification of healthcare services, developing and adopting interoperability standards for health data exchange across borders, and implementing consumer protection measures to ensure that patients are informed about the risks and benefits would help to address some of the concerns about patients receiving safe, effective, and high-quality care across borders. One well-known example is the Asia-Pacific Economic Cooperation (APEC) Cross-Border Privacy Rules (CBPR), which facilitates the transfer of personal data across APEC member economies while ensuring privacy protections, serving as a model for data governance in healthcare AI [43].

### 3.5. Ethics and Equitable Access

Regulators have to ensure that patient data collection, sharing, and usage are governed by underlying ethical principles [6,44]. As the use of LLMs increases, unethical use, such as spread of misinformation becomes a major risk, especially in the healthcare context. Hence, the intention of the AI, distinction between medical and non-medical datasets, and equity must be addressed by regulators.

#### 3.5.1. Intent of AI Design

Developers of AI technology are encouraged to generate their own ethics guidelines to avoid harm, such as violations of human rights or bodily injury, and many companies do set out to provide such norms and standards. However, in many instances, this appears to be “ethics washing” as the developers’ guidelines often do not address causal responsibility or retrospective harm [45]. Furthermore, monitoring their adherence to those guidance documents is not transparent and is also performed internally, so that even if they are not met, there is no legal enforcement or consequence [45].

#### 3.5.2. Distinction between Medical and Non-Medical Data

In the context of the patients and users of AI technologies, wearable devices are extensively being used to monitor “healthy” individuals and large amounts of data are being collected through them. Ethical concerns arise when it comes to usage of such data for non-medical purposes, such as insurance companies using these to determine premiums.

Therefore, non-medical data may also need to be protected similar to data protection in formal clinical care settings. Non-medical data encompasses information other than an individual’s health, medical conditions, treatments, and outcomes. It may include demographic data, behaviours, financial information, employment history, social media activities, and personal preferences. Regulators would need to provide frameworks on distinguishing data being used for medical and non-medical objectives.

#### 3.5.3. Equity

In the context of ethics and equitable access, it may be tricky to fulfil certain fundamental rights. For example, the right to non-discrimination prohibits distinguishing people based on race; however, if variables such as race are ignored while training AI systems, this may lead to erroneous results and bias. Hence, in AI systems, as expressed by Cohen et al., in their article, it is important to factor in all demographic factors, irrespective of the fundamental rights so that the outputs produced by the AI system are equitable to all people [46].

#### 3.5.4. Recommendations

The first step of generating their own ethics guidelines by developers of AI technology is already in place. Adherence to these guidelines could be monitored by other developers in the same field or by regulators. Certifications or recognitions could be awarded to developers who strictly abide by their ethics guidelines. Such recognition could create a positive situation where developers apply and advertise their use of such guidelines, while regulators can promote and highlight the application of these guidelines through an appropriate monitoring framework that enlists other players in the field.

It is essential to engage with local communities and foster collaboration between public and private sectors, as well as among healthcare providers, researchers, and technology companies to help develop AI solutions that are both effective and accessible to all, irrespective of their socio-economic status. Health equity impact assessments can be conducted to evaluate the potential impact of AI technologies on different populations. This can help in identifying and addressing any disparities that may arise.

## 4. Limitations of This Study

The recommendations provided for each of the gaps identified may not be extensive and may also have their own limitations. The recommendations may also need to be adapted to fit the specific healthcare system and regulatory environment of each region. In countries with centralised healthcare systems, regulations might focus on nationwide standards and government oversight, ensuring uniformity and consistency across the system. In contrast, in decentralised or privatised healthcare systems, regulations may need to account for variability across providers and incorporate more flexible guidelines that allow for local organisational discretion. Additionally, regions with emerging healthcare markets might require tailored regulations that balance innovation with resource constraints, while more established markets may emphasise rigorous compliance and advanced patient protection standards. Further, as the field evolves, it may be necessary to revisit the five themes covered here and consider additional areas that may require regulatory attention.

## 5. Conclusions

As countries around the world are working toward regulating AI-based health systems using the total product lifecycle and high-risk approaches, it is essential to address the regulatory gaps highlighted under the five major thematic issues of concern.

To ensure ***data security and protection***, the use of data needs to be defined and limited, and patients should be given full liberty encompassing the ability to erase their stored data to stop further usage. Regulations pertaining to personal data protection need to be considered, including anonymized data and stringent requirements that can be put in place to minimise the risk of the re-identification of patient data.

It is crucial to ensure that the ***quality of data*** that is used to train algorithms fulfils the four properties of variety, volume, veracity, and velocity. Disclosing the attributes of the training data would also enable ***validating the AI algorithms***. It is also important to address the black box challenge through various explainable AI techniques to provide clarity to physicians on what basis the decisions or recommendations were made by the AI systems. More research in the areas of saliency maps coupled with natural language processing would facilitate this. For continuous learning models, rigorous testing and validation protocols along with ongoing frequent monitoring and evaluation of the model performance would facilitate their safety and effectiveness.

To help address ***accountability concerns***, establishing mutual recognition agreements across borders for licencing and certification of healthcare services and adopting interoperability standards for safe health data exchange would help to resolve cross-border issues of AI in healthcare services. Finally, to address the challenges pertaining to ***ethics and equitable access***, certifications or suitable recognitions could be considered for developers who abide by their own ethics guidelines. Regular health equity impact assessments would enable addressing disparities and help ensure that employing AI in healthcare services not only benefits select groups but whole populations.

## Figures and Tables

**Table 1:** Overview of key AI challenges in data security and protection.

Challenges	Underlying Issues
**Anonymisation of data**	Personal data protection regulations are not applicable for anonymised data. However, with advancements in technology, it is possible to re-identify patients even when their data have been anonymised.
**Data exportation**	Due to lack of uniform data protection across different jurisdictions, there is a possibility of companies developing AI models to obtain data from countries with weak or no data protection regulations, and a compromise in the security and protection of such data that may not be sufficiently governed by regulatory frameworks.
**Informed Consent**	Patients and users should be informed and give consent when using AI/ML applications. They should understand and decide on the details that they wish to disclose but this is generally not adequately addressed by existing governance frameworks.

**Table 2:** Overview of key challenges for validating AI algorithms.

Challenges	Underlying Issues
Validation of algorithms	The process of checking AI algorithms to ensure that the requirements, specifications and intended purpose are met is difficult [29]
Black box nature of AI models	An algorithm that self-learns by continuously testing and adapting to its own analysis procedure is a black box algorithm. While AI can be used in diagnostic procedures, for example, detecting pathologies in X-ray images, the process by which the underlying algorithm reaches its diagnosis cannot be accounted for by physicians, raising problems in trusting the diagnosis when it cannot be determined how it was made [30]
Explainability	The ability to understand how a particular decision was made is difficult for the physicians [3]
Causability	The measurement of the quality of the explanations by the AI models is currently not possible [31]

## Data Availability

Not applicable.
